# SAM-Based Approach for Automated Fabric Anisotropy Quantification in Concrete Aggregates

**DOI:** 10.3390/s25216661

**Published:** 2025-11-01

**Authors:** Zongxian Liu, Chen Chen, Huibao Huang, Jiankang Chen, Pengtao Zhang, Jianghan Xue

**Affiliations:** 1 State Key Laboratory of Hydraulics and Mountain River Engineering, College of Water Resources & Hydro-Power, Sichuan University, Chengdu 610065, China; liuzongxian@stu.scu.edu.cn (Z.L.); huanghuibao@stu.scu.edu.cn (H.H.); scu-jiankang@scu.edu.cn (J.C.); zhangpengtao@stu.scu.edu.cn (P.Z.); xuejianghan@stu.scu.edu.cn (J.X.); 2 Yalong River Valley Hydropower Development Co., Ltd., Chengdu 610051, China

**Keywords:** concrete aggregate, segment anything model (SAM), fabric anisotropy, image process, segmentation

## Abstract

The reliable characterization of fabric anisotropy in concrete aggregates is critical for understanding the mechanical behavior and durability of concrete. The accurate segmentation of aggregates is essential for anisotropy assessment. However, conventional threshold-based segmentation methods exhibit high sensitivity to noise, while deep learning approaches are often constrained by the scarcity of annotated data. To address these challenges, this study introduces the Segment Anything Model (SAM) for automated aggregate segmentation, leveraging its remarkable zero-shot generalization capabilities. In addition, a novel quantification technique integrating computational geometry with second-order Fourier series is proposed to evaluate both the magnitude and orientation of fabric anisotropy. Extensive experiments conducted on a self-constructed concrete aggregate dataset demonstrated the effectiveness and accuracy of the proposed method. The process incorporates domain-specific image preprocessing using Contrast Limited Adaptive Histogram Equalization (CLAHE) to enhance the input quality for the SAM. The SAM achieves an *F*_1_-*score* of 0.842 and an intersection over union (*IoU*) of 0.739, with mean absolute errors of 4.15° for the orientation and 0.025 for the fabric anisotropy. Notably, optimal segmentation performance is observed when the SAM’s grid point parameter is set to 32. These results validate the proposed method as a robust, accurate, and automated solution for quantifying concrete aggregate anisotropy, providing a powerful tool for microstructure analysis and performance prediction.

## 1. Introduction

Concrete dominates civil engineering infrastructure due to its exceptional compressive strength, durability, versatility, and cost efficiency, serving as a fundamental material for buildings, bridges, dams, roads, and tunnels [1]. The macroscopic mechanical behavior of concrete is critically governed by its structural characteristics, with aggregate properties such as spatial distribution, morphology, and orientation affecting its strength, deformation, fracture, and long-term durability [2,3]. Although extensive research has examined the effects of aggregate distribution and morphology, aggregate orientation remains largely unquantified [4]. Orientation refers to the alignment of aggregate particles within hardened concrete and is a fundamental factor governing fabric anisotropy. It is inherently random due to casting vibrations and interactions with mortar and surrounding aggregates [5] and serves as a key structural indicator for assessing the fabric anisotropy and predicting concrete behavior [6,7]. Therefore, quantification of aggregate orientation is essential for relating microstructural features to concrete performance [8].

Accurate evaluation of aggregate orientation has traditionally relied on destructive sampling, such as coring or cross-sectioning, followed by visual inspection to determine the aggregate direction and distribution [5]. These manual approaches are subjective, time-consuming, and error-prone, limiting the precise quantification of aggregate anisotropy. Image processing techniques have therefore emerged as effective tools for automated evaluation, typically categorized into indirect and direct methods. Indirect approaches first segment individual particles from images [9] and then extract orientations through geometric analysis to quantify the material anisotropy [10]. Conventional segmentation methods, including watershed segmentation, thresholding, and edge detection, separate images into distinct regions based on features such as grayscale, texture, and shape. For example, Wang et al. [6] proposed image-processing methods to identify aggregates and their boundaries, obtaining orientations by connecting the image center with each aggregate center. Zhang et al. [11] utilized the ImageJ software to recognize each aggregate with surrounding rectangles. Han et al. [8] combined grayscale-based identification with manual correction. These methods, however, often require manual parameter inversion and are sensitive to image noise, limiting the accuracy and efficiency, particularly for complex two-dimensional granular images. In contrast, direct analytical approaches extract particle anisotropy directly from image features without the need for segmentation [12,13,14]. The rotational Haar wavelet transform (RHWT) has been widely applied to quantify fabric anisotropy and orientation in various materials, including sands [15], high explosives [16], and scanning electron microscope images of cemented sand [17]. The RHWT analyzes directional variations in boundary pixels to determine particle orientations, but its applicability and accuracy for concrete aggregates remain to be fully validated.

Recent advances in artificial intelligence, particularly deep learning, have opened new opportunities for the quantitative analysis of concrete. Classical deep convolutional neural network-based classical segmentation algorithms, such as fully convolutional neural network (FCN) [18], U-Net [19], and mask region-based convolutional neural network (Mask R-CNN) [20], have achieved notable success in tasks such as crack segmentation [21], aggregate identification [22], and damage assessment [23,24]. For instance, Chow et al. [25] applied U-Net to segment kaolinite particles in clay and quantified their directional distribution using the fabric tensor, with orientations extracted from the major particle axes. Wang et al. [26] developed an enhanced DeepLabv3+ framework with SE-block-modified ResNeXt50, achieving concrete aggregate segmentation for stability assessment. However, deep learning methods for concrete aggregate anisotropy are limited by high resource demands and reliance on domain expertise. Annotated images require destructive sampling and labor-intensive labeling, while model development depends on specialized knowledge, creating barriers to widespread use. These challenges highlight the need for automated methods that reduce manual effort while maintaining accuracy.

To address the limitations of existing methods for evaluating concrete aggregate anisotropy, this study develops a computational approach based on the Segment Anything Model (SAM) for automated quantification of aggregate orientation and fabric anisotropy. The main contributions are as follows: (1) We present a four-module computational pipeline integrating (i) domain-specific image preprocessing using Contrast Limited Adaptive Histogram Equalization (CLAHE), (ii) zero-shot segmentation of concrete aggregates using the SAM, (iii) extraction of individual aggregate orientations via computational geometry-based fitting, and (iv) computation of fabric anisotropy using the fabric tensor. (2) We incorporate second-order Fourier analysis to evaluate the dominant directional trend within each image, thereby refining the anisotropy characterization. (3) The proposed approach is validated on a self-constructed dataset of concrete aggregate images. The results show that it enables accurate, automated quantification of aggregate anisotropy and provides a potential tool for linking microstructural features to concrete performance.

## 2. Methodology

### 2.1. Image Preprocessing Using CLAHE

The primary objective of the CLAHE [27] algorithm is to improve the contrast of an image while reducing the noise. It accomplishes this by adjusting the contrast in a way that enhances the overall quality and clarity of the image. Additionally, the algorithm has the benefit of preserving fine details and controlling the level of contrast.

The necessity of CLAHE preprocessing stems from two domain-specific challenges in concrete imaging: (1) non-uniform illumination artifacts induced by wet-drilling residue create localized shadows that obscure aggregate boundaries, which cannot be fully compensated for by deep learning’s normalization layers; (2) low-contrast texture similarity between aggregate and mortar regions causes ambiguous edge transitions, requiring targeted enhancement to amplify discriminative features.

**Figure 1:**
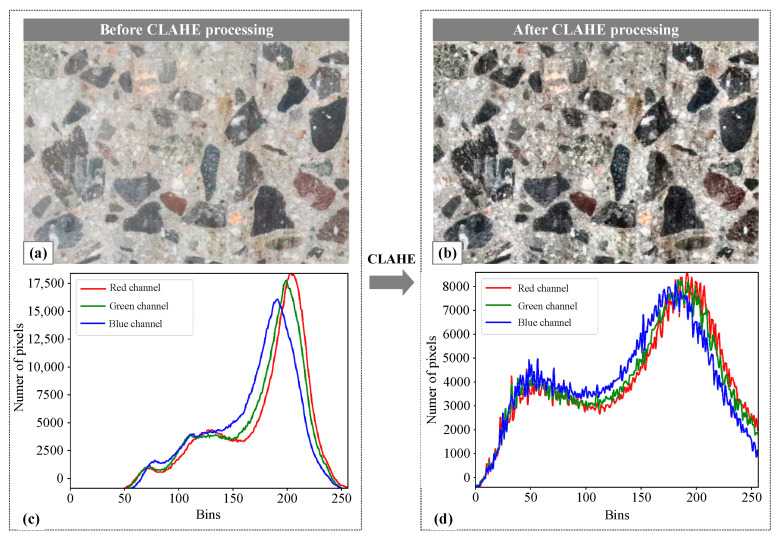
Concrete image processing based on the CLAHE algorithm: (**a**) original image; (**b**) image processed using the CLAHE algorithm; (**c**) quantitative analysis of RGB values in the original image; (**d**) quantitative analysis of RGB values in the processed image.

The images obtained from core drilling often have reduced contrast due to the equipment’s cutting and friction. This can lead to unclear details, information loss, and decreased visual quality. To address this issue, the CLAHE algorithm is used to enhance the contrast by adjusting the brightness in different regions of the image based on local features. This improves the visibility of concrete aggregates and mortar, resulting in better visual quality. The adaptive enhancement provided by the algorithm is crucial for accurately extracting detailed edge features of aggregate particles. Additionally, the CLAHE algorithm includes contrast limitations to prevent excessive enhancement of noise and local details during the equalization process. This not only preserves image details but also improves the overall visual quality. As such, the CLAHE algorithm was utilized in this study to improve the contrast of the original concrete image and minimize the impact of noise. Figure 1 displays the cross-sectional image of hardened concrete. The original image (Figure 1a) was subjected to the CLAHE algorithm, resulting in a more pronounced contrast in Figure 1b that emphasized local details. Furthermore, a quantitative analysis of the RGB channels in the processed image indicate a decrease in peak intensity from 17,500 (Figure 1c) to approximately 8000 (Figure 1d), suggesting a more balanced image after processing.

### 2.2. SAM-Based Concrete Aggregate Segmentation

The SAM [28] represents a comprehensive deep learning vision model designed as a versatile tool for image segmentation. It has undergone extensive training on an extensive dataset, encompassing over one billion masks across eleven million images. Consequently, the SAM demonstrates remarkable zero-shot generalization capabilities to unexpected data, eliminating the necessity for additional training. Because of its outstanding performance across various computer vision benchmarks, the SAM has garnered significant attention for segmentation tasks [29], such as in medical imaging [30], civil engineering [31], remote sensing [32], and industrial engineering [33].

From a theoretical perspective, the suitability of the SAM for concrete aggregate segmentation lies in three aspects. First, its ViT-based encoder extracts robust global features, enabling accurate delineation of aggregates with heterogeneous textures and overlapping boundaries. Second, its flexible prompt encoder allows integration of sparse or dense guidance, which enhances the segmentation consistency under diverse imaging conditions. Third, the SAM’s lightweight mask decoder ensures efficient inference, which is beneficial for large-scale analysis of aggregate images. This characteristic aligns with concrete aggregate segmentation needs, where morphological isolation suffices for geometric analysis, making the SAM exceptionally suitable for this study’s focus. Consequently, this study explores the SAM’s performance when identifying concrete aggregates.

**Figure 2:**
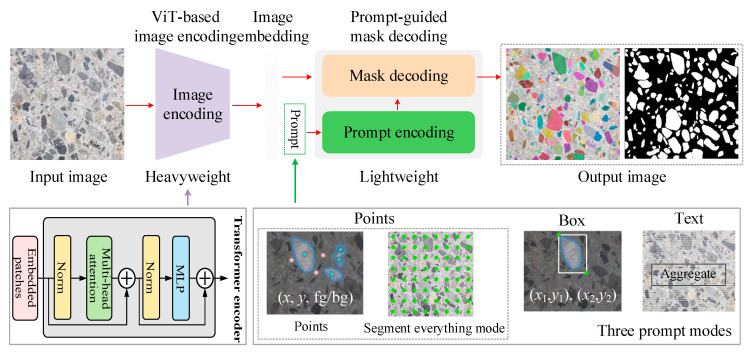
The overview of SAM for concrete aggregate segmentation.

The SAM’s structure comprises three essential components, namely, a Vision transformer (ViT)-based image encoder (*F*_i-enc_), a prompt encoder (*F*_p-enc_), and a lightweight mask decoder (*F*_m-dec_). The schematic diagram of the SAM is illustrated in Figure 2. The *F*_i-enc_ section relies upon a conventional ViT that has undergone prior pre-training using the Masked Autoencoder (MAE) methodology for image feature extraction. The outcome of the image-encoding process is a 16× downsampled embedding of the input image. The *F*_p-enc_ provides the positional information for the *F*_m-dec_ through the utilization of interactive input points, boxes, and text. In this work, the “everything” mode was selected to automatically generate masks on almost every aggregate. The *F*_m-dec_ is a lightweight decoder with a dynamic mask prediction head that incorporates the embedded prompt and the image embedding from *F*_i-enc_. The main mathematical model can be expressed as Equation (1):

(1) $$\left\{\begin{array}{l} F_{\mathrm{img}}=F_{i-\mathrm{enc}}(I)\\ T_{\mathrm{pro}}=F_{p-\mathrm{enc}}(\{p\})\\ O_{\mathrm{pre}}=F_{m-\mathrm{dec}}(F_{\mathrm{img}}+F_{c-\mathrm{mask}},[F_{\mathrm{out}},T_{\mathrm{pro}}]) \end{array}\right.$$

where $I\in {\mathbb{R}}^{H\times W\times 3}$ represents the input image, and *H* and *W* are the image height and width, respectively. *F*_img_ denotes the intermediate features that are extracted by the ViT module. {*p*} represents either sparse prompts (e.g., points, boxes, text) or dense prompts (masks), while *T*_pro_ denotes the corresponding prompt tokens encoded by *F*_p-enc_. *F*_c-mask_ denotes the optional input with a coarse mask for the SAM, and *F*_out_ denotes the learnable tokens from different masks and their corresponding intersection over union (*IoU*) predictions. *O*_pre_ denotes the predicted masks.

As this work eliminated the need to differentiate aggregate types, we employed the SAM in the “everything” mode to generate candidate masks across the entire image, ensuring that nearly all aggregates were captured. The resulting binary masks provided reliable input for downstream tasks, including the orientation and anisotropy quantification. This pipeline design leverages the SAM’s broad generalization capacity while incorporating lightweight, domain-specific refinements, thereby ensuring both accuracy and efficiency during aggregate segmentation.

### 2.3. Aggregate Orientation Extraction

**Figure 3:**
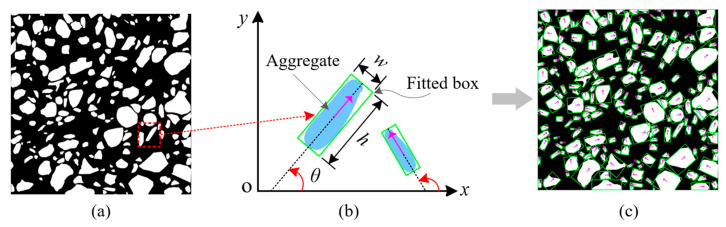
Orientation fitting of each concrete aggregate: (**a**) aggregate segmentation; (**b**) aggregate orientation; (**c**) orientation fitting.

Based on the SAM segmentation results from delineating individual concrete aggregates (Figure 3a), the process for extracting the aggregate orientation involves the following key steps: (1) The contour extraction algorithm is employed to capture the continuous pixel coordinates outlining the aggregate particles’ boundaries, which effectively represents their shapes. (2) For each contour, image-processing techniques are applied to determine the minimum bounding rectangle that fully encloses the contour while minimizing its area, which is referred to as the smallest external rectangle. (3) Regarding a set of points *P* = {*p_k_* (*x_k_*, *y_k_*), *k* = 1, 2, 3, 4}, the algorithm computes the covariance matrix and performs eigenvalue decomposition on the points *P*. The eigenvector associated with the largest eigenvalue of *P* signifies the major axis direction of the smallest external rectangle, and this direction represents the orientation of the aggregate particles.

The aggregate direction is defined as depicted in Figure 3b. For each aggregate *i*, the minimum external rectangle is characterized by dimensions *w* × *h*, and the angle between its major axis direction and the horizontal *x*-axis is identified as the particle’s orientation *θ*. The aggregate direction *θ* varies between 0° and 180°. The minimum external rectangle and direction-fitting results of individual particles are illustrated in Figure 3c. Computer vision techniques effectively extract aggregate orientation information from input images, establishing essential groundwork for subsequent anisotropy analysis.

### 2.4. Fabric Anisotropy Analysis

The quantification of granular material fabric anisotropy can be achieved using a fabric tensor. Within this theoretical framework [34,35], we define an evaluation index for concrete fabric anisotropy, which is derived from the orientation of aggregate particles in two-dimensional space, and expressed as Equation (2):

(2) $$\Delta =\frac{1}{N}\left[{\left(\sum_{i=1}^{N}\cos2\theta_{i}\right)}^{2}+{\left(\sum_{i=1}^{N}\sin2\theta_{i}\right)}^{2}\right]$$

where Δ quantifies the degree of fabric anisotropy, *θ_i_* is the orientation of *i*-th aggregate (Figure 3b), and *N* represents the total number of aggregate particles. As such, the value of Δ ranges from 0 when the aggregate particles have a completely random particle orientation distribution to 1 when all the long axes of the aggregate particles are in the same direction.

Considering the impact of segmentation errors, image noise, and local aggregate display at image edges, the second-order Fourier series [17] is utilized to fit the distribution of the aggregate particle direction. The fitting function *F*(*θ*) can be expressed as Equation (3):

(3) $$F\left(\theta \right)=a_{0}+a_{1}\cos\left(\theta \right)+b_{1}\sin\left(\theta \right)+a_{2}\cos\left(2\theta \right)+b_{2}\sin\left(2\theta \right)$$

**Figure 4:**
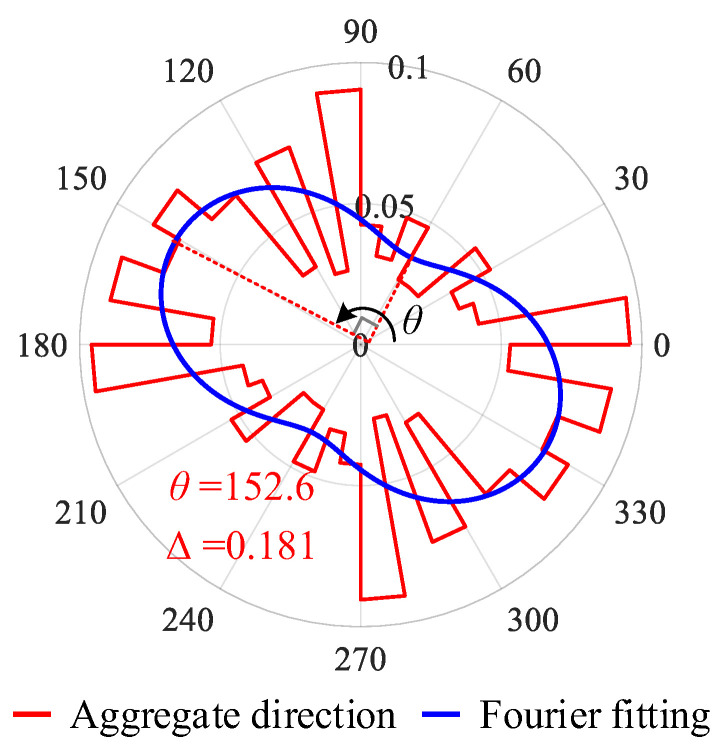
Results in the polar coordinate system of the concrete aggregate direction.

Due to the symmetry (i.e., *F*(θ + 180°) = *F*(θ)) in Equation (3), *a*_1_ and *b*_1_ must be zero. Thus, Equation (3) can be expressed as:

(4) $$\begin{array}{ll} F\left(\theta \right) & =a_{0}+a_{2}\cos\left(2\theta \right)+b_{2}\sin\left(2\theta \right)\\ \; & =a_{0}+\sqrt{a_{2}^{2}+b_{2}^{2}}\sin(\beta +2\theta ) \end{array}$$

where $\sin\beta =\frac{a_{2}}{\sqrt{a_{2}^{2}+b_{2}^{2}}}$ and $\cos\beta =\frac{b_{2}}{\sqrt{a_{2}^{2}+b_{2}^{2}}}$. The coefficients *a*_0_, *a*_2_, and *b*_2_ can be determined by fitting Equation (4) according to the original aggregate direction plot (Figure 4). *F*_max_ and *F*_min_ are computed using Equation (5):

(5) $$\left[\begin{array}{c} F_{\max}\\ F_{\min} \end{array}\right]=a_{0}\pm \sqrt{a_{2}^{2}+b_{2}^{2}}$$

Taking Figure 3a as an example, the statistical direction of aggregates in the image and the Fourier fitting results are shown in Figure 4. Among them, the direction statistics of the concrete aggregate are presented as the red curve, utilizing 10 degrees as the unit interval. Then, *a*_0_, *a*_2_, and *b*_2_ could be computed as 0.05556, 0.01203, and −0.01711, respectively. Thus, *F*_max_ = 0.076 and *F*_min_ = 0.035 were computed according to Equation (5), and the fabric direction *θ* (direction of *F*_max_) could be computed as 152.6° and Δ could be computed as 0.181 using Equation (2).

## 3. Dataset

The experiments were conducted using self-constructed datasets. The concrete images were captured from the structural components with specific concrete compositions outlined in Table 1. This mix proportion was selected for its conformity with standard C30-grade structural concrete commonly used in hydro-power engineering. It reflects established engineering practices, ensuring relevance to typical field conditions. The aggregates of concrete primarily comprised fine and coarse aggregates, with a composition ratio of 35.6% for fine aggregates and 11.9% for coarse aggregates. The coarse aggregates predominantly fell within the 10–20 mm size range.

To establish the image analysis database, the following procedure was adopted: (1) Specimen preparation: After the concrete achieved its design strength and hardening, structural sections were extracted using a cutting machine to expose representative cross-sections. (2) Surface treatment: Each cross-section was carefully rinsed with water to remove debris and surface artifacts from the cutting process. (3) Image acquisition: High-resolution photographs were captured using a SONY A6000 camera (Sony Corporation, Wuxi, China) positioned orthogonally 200–400 mm away under controlled lighting. The original images had a resolution of 4000 × 6000 pixels. (4) Pre-processing: The raw images were cropped into standardized 500 × 500-pixel patches to ensure consistency across the dataset. (5) Annotation: Manual labeling was conducted using the Labelme software (version 5.7.0, https://github.com/wkentaro/labelme, accessed on 15 March 2025) to generate ground truth masks, where aggregates were represented by white pixels and mortar by black pixels. The dataset includes aggregates of varying sizes, from fine aggregates (5–10 mm) and coarse aggregates (10–20 mm). This scale ensures that the dataset is directly applicable to practical engineering scenarios, providing a solid foundation for the development and application of the anisotropy index in real-world concrete design.

**Figure 5:**
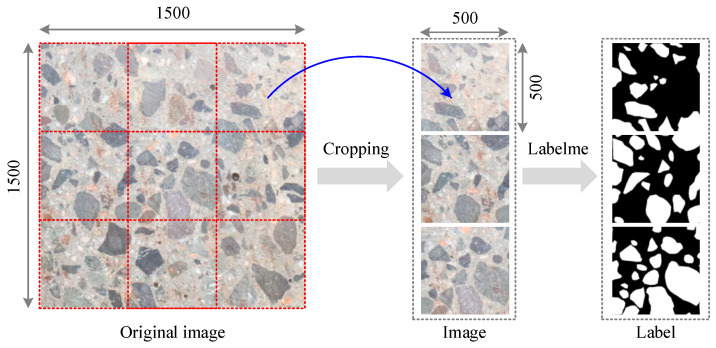
Concrete aggregate image acquisition and label.

Following this procedure, a total of 108 image–label pairs were obtained to evaluate the SAM’s segmentation performance. Figure 5 presents representative examples of the raw images and their corresponding ground truth labels.

## 4. Experiment Results and Discussion

This study was conducted on a single NVIDIA RTX 3060 (NVIDIA Corporation, Shenzhen, China) graphics processing unit with 12 GB. The SAM (https://github.com/facebookresearch/segment-anything, accessed on 20 March 2025) was encoded using Python (version 3.9) language and implemented with PyTorch (version 1.10.0) framework. The official checkpoint of the ViT-H SAM was selected to evaluate the optimal performance of SAM in the context of zero-shot concrete aggregate segmentation. The “everything” mode in SAM, which can prompt the SAM using many points distributed across the image to automatically segment almost every aggregate, was selected.

The key experimental parameters are detailed as follows: CLAHE preprocessing was applied with a clip limit of 2.0 and a grid size of 8 × 8. SAM parameters were configured with a Predict *IoU* threshold of 0.96, stability score threshold of 0.95 (offset 0.95), confidence threshold of 0.9, minimum mask region area of 100 pixels, and crop number of layers of 1. In addition, the grid points were set as to 32 during inference.

### 4.1. Evaluation Metrics

Four quantitative metrics are introduced for the comprehensive evaluation of different segmentation models, namely *Precision*, *Recall*, *F*_1_-*score*, and *IoU*. To assess the concrete aggregate segmentation accuracy, a comparative analysis was conducted against the annotated mask (Figure 5). Specifically, the aggregate pixels with white color are considered as positive instances and the suspension pixels with black color are considered as negative instances. Precision quantifies the proportion of accurately classified aggregate pixels to the total number of classified aggregates. Conversely, recall quantifies the proportion of all the aggregate pixels that are correctly classified. A high precision indicates a high accuracy of the aggregate detection results, while a high recall indicates that fewer aggregate pixels are missed during the detection process. The *F*_1_-*score* takes into account both the precision and recall of the classification model. IoU captures the intersection of the union between the predicted segmentation and the ground truth. The four evaluation indicators are defined as follows:

(6) $$Precision=\frac{TP}{TP+FP}$$

(7) $$Recall=\frac{TP}{TP+FN}$$

(8) $$F_{1}-score=2\times \frac{Precision\times Recall}{Precision+Recall}$$

(9) $$IoU=\frac{TP}{TP+FP+FN}$$

where true positive (*TP*) is defined as the aggregate pixels that are accurately identified, false positive (*FP*) is the incorrectly detected aggregate pixels, and false negative (*FN*) is the aggregate pixels that remain undetected.

For the evaluation of the segmentation performance, *Precision*, *Recall*, and *IoU* were calculated on a per-image basis using the binary masks of concrete aggregates, with the ground truth labels serving as a reference. Specifically, each predicted segmentation was compared against the manually annotated binary mask to quantify the correctly and incorrectly classified aggregate pixels. The resulting metrics were then averaged uniformly across the entire test set, so that each sample contributed equally to the overall performance. This averaging strategy ensured that the reported values reflect the robustness of the method across diverse images and avoids bias toward images containing a larger number of aggregates.

### 4.2. Comparison of SAM with Other Segmentation Methods

**Figure 6:**
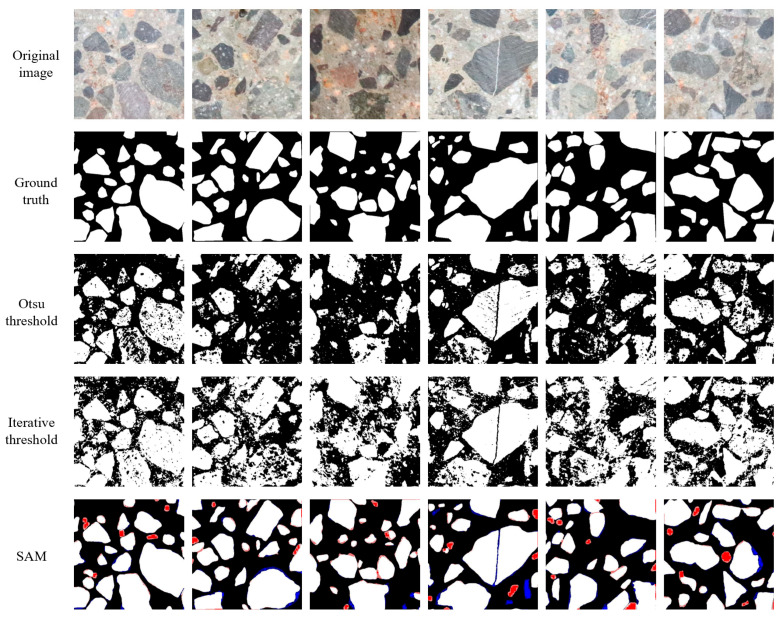
Comparison of segmentation results obtained using different methods. Note: White, red, and blue denote true positive, false positive, and false negative pixels, respectively.

To comprehensively evaluate the SAM’s segmentation performance, the traditional classical Otsu thresholding and iterative thresholding segmentation methods were selected for comparison. They can directly extract the shallow features of the image without additional training process, and achieve the best segmentation performance of aggregate and mortar through the most suitable threshold. The performance evaluation of diverse segmentation methods is presented in Table 2, and some segmentation samples are demonstrated in Figure 6. Traditional segmentation approaches struggle with complex aggregate image segmentation, yielding noisy outcomes and exhibiting blurred aggregate contours. The evaluation index *IoU* indicates the suboptimal performance of classical segmentation techniques in dealing with intricate images. The segmentation metrics *Precision*, *Recall*, *F*_1_-*score*, and *IoU* of the SAM are 0.894, 0.796, 0.842, and 0.739, respectively. The results demonstrate that the SAM achieved significant improvement regarding performance and achieved impressive capacity for zero-shot generalization, demonstrating its potential for robust performance across diverse scenarios. Notably, precision surpasses recall, indicating that the positive class (i.e., concrete aggregates) is correctly predicted, but some of the mortar class is also been erroneously classified as aggregates. That is, the SAM can accurately identify the aggregate class while maintaining a low probability of missing concrete aggregates. It is worth mentioning that some mortar may be misclassified as aggregates due to the small size of the fine aggregate, which may not be explicitly treated as aggregates during manual annotation. However, SAM could effectively capture subtle boundary pixel changes between aggregates and mortar, ensuring accurate classification of the aggregate category, as evidenced by the SAM’s segmentation results in Figure 6.

### 4.3. Effect of Grid Points on SAM Performance

**Figure 7:**
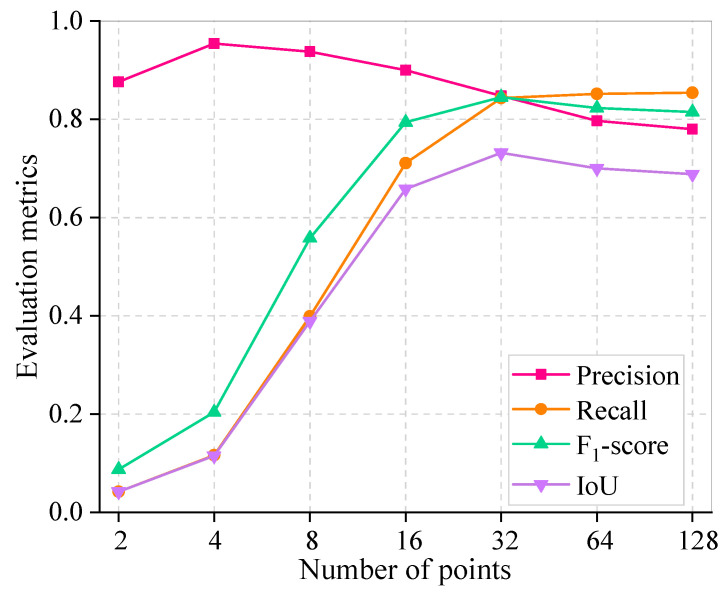
Sensitivity analysis of different numbers of grid points.

**Figure 8:**
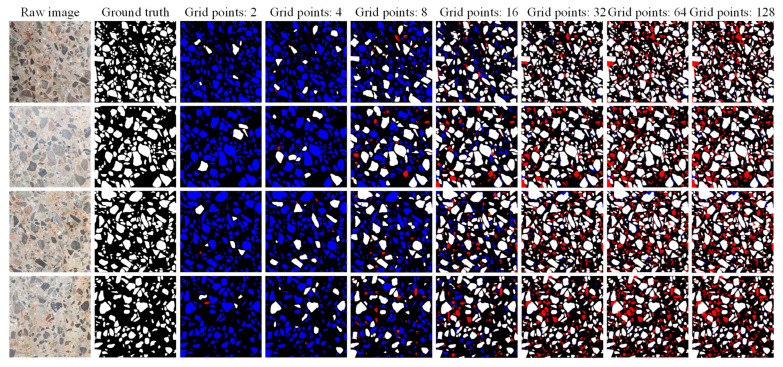
Segmentation examples of different numbers of grid points. Note: True positive pixels are shown in white, false positive pixels are shown in red, and false negative pixels are shown in blue.

Table 3 and Figure 7 demonstrate the SAM’s segmentation performance with different grid points and their corresponding computation time. The image pixels used in the experiment were 1500 × 1500 to better represent the performance of different grid points on image segmentation. The evaluation metrics recall, *F*_1_-*score*, and *IoU* show a significant increasing trend with the increase in grid points, which reaches the peak with a point setting of 32 and subsequently experiences a slight decreasing trend. This indicates that denser grid points do not lead to a superior performance. This phenomenon is chiefly attributed to the fact that the SAM’s optimal segmentation performance is not achieved with fewer grid points, and too many grid points lead to over-segmentation. Partial image segmentation for different grid point scenarios is depicted in Figure 8. This is corroborated by a discernible progression in segmentation performance, transitioning from under-segmentation (indicated by blue pixels) to over-segmentation (indicated by red pixels) with the increasing number of grid points. Furthermore, the precision is higher in general, and an explanation for this phenomenon is provided in Section 4.2. Furthermore, it is noteworthy that an increase in the number of grid points proportionally extends the computational time for an image with 1500 × 1500 pixels. Doubling the number of grid points (greater than 16) essentially increases the computational time by a factor of 2. As such, setting the grid point to 32 in this work is reasonable.

### 4.4. Evaluation of Concrete Aggregate Anisotropy

Illustrated using four distinct images with a resolution of 3000 × 1500 pixels, the proposed methodology commenced with the application of CLAHE processing to enhance the image quality. Subsequently, the SAM-based segmentation was executed. Considering the uniform generation of grid points in “segment everything mode”, the 3000 × 1500-pixel image was initially decomposed into 1500 × 1500-pixel sub-regions. These sub-regions were subsequently concatenated after SAM segmentation. A comprehensive performance evaluation of image segmentation based on SAM is summarized in Table 4. The average values of evaluation metrics *Precision*, *Recall*, *F*_1_-*score*, and *IoU* are 0.919, 0.839, 0.877, and 0.781, respectively. These results verified the superior accuracy and generalization capabilities of the SAM in the concrete aggregate segmentation context.

**Figure 9:**
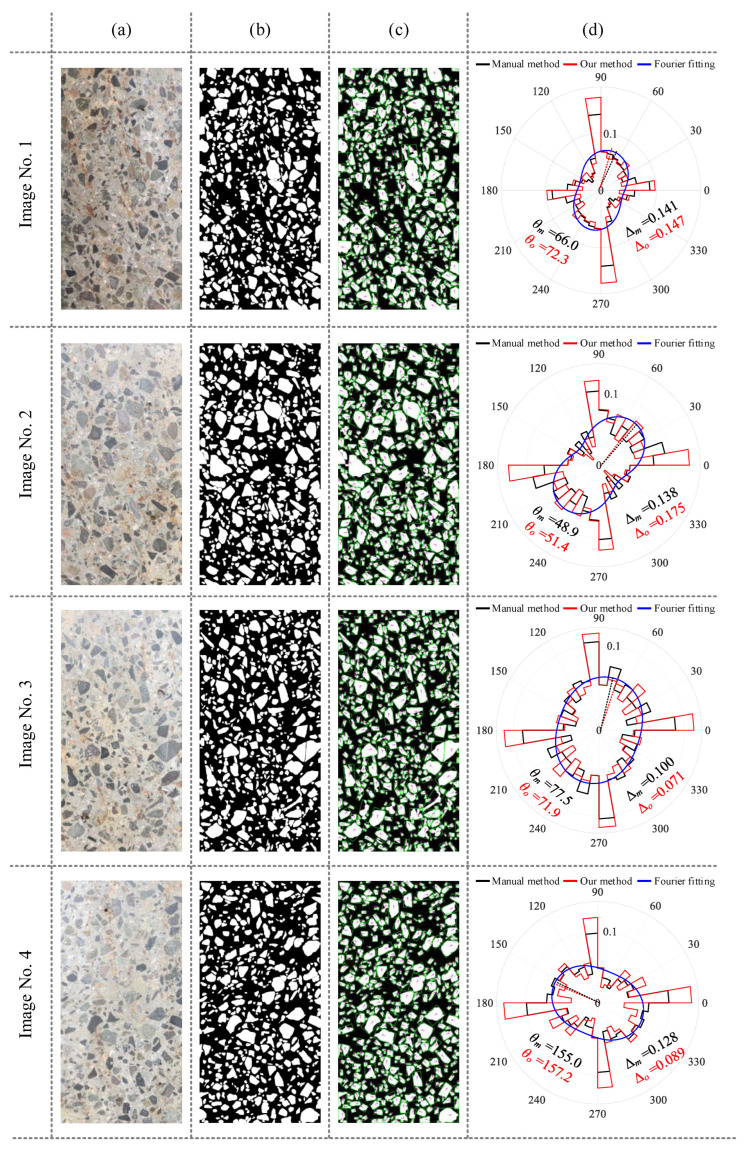
Computational results for different concrete images: (**a**) original image; (**b**) aggregate segmentation; (**c**) orientation fitting; (**d**) anisotropy evaluation.

After image segmentation was completed, the aggregate directions were determined and fitted through the application of minimum bounding rectangles. The segmentation results of four sample images, the fitted aggregate directions, and the rose plot of aggregate direction are shown in Figure 9. Among them, manual annotation, based on manual assessment of aggregate orientations, is included and compared with proposed method to validate its efficacy. A comprehensive evaluation of aggregate direction and anisotropy obtained from four images is provided in Table 5. The results indicate that the maximum error, minimum error, and average values of the angles are 6.3°, 2.2°, and 4.15°, respectively. The maximum, minimum, and average values of fabric anisotropy indicators are 0.039, 0.006, and 0.025, respectively. Figure 9 illustrates an abundance of aggregates oriented near 0° and 90°. This phenomenon can be attributed to two factors. First, the prevalence of nearly circular particles results in similar dimensions for length and width during aggregate fitting, leading to orientations near 0° or 90°. Second, due to image boundary constraints, aggregates located at the boundary exhibit straight edges, thereby favoring orientations approximating 0° or 90° during the fitting process.

**Figure 10:**
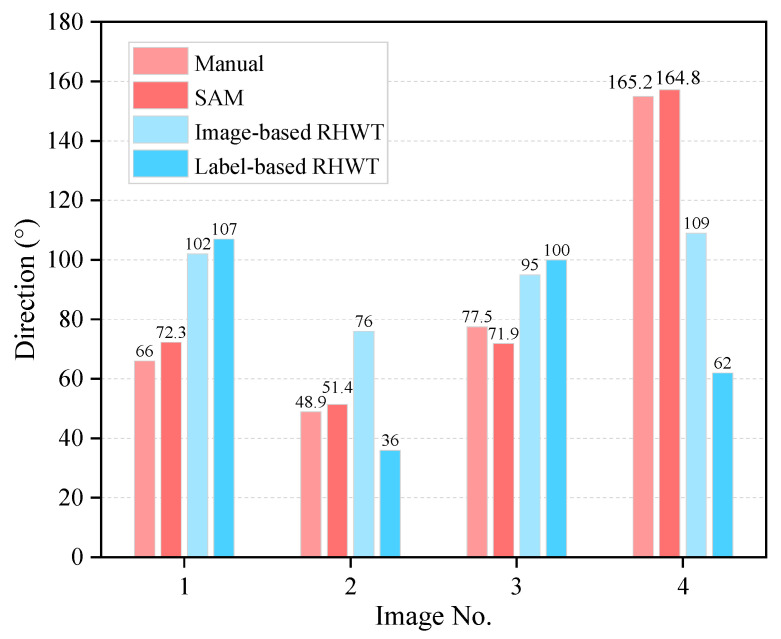
Direction evaluation results of different methods for concrete aggregate.

The results of concrete aggregate direction evaluation using different methods are illustrated in Figure 10. The RHWT method is utilized to evaluate the direction of the original image and segmented label image. The results indicate a discernible disparity between the outcomes derived from the RHWT method and this work. The potential cause may be the image noise in the RHWT evaluation results, and the RHWT method’s applicability for concrete aggregate direction may need further validation.

### 4.5. Limitations and Future Work

This study demonstrates the feasibility of combining an unsupervised SAM-based segmentation framework with a Fourier descriptor to quantify fabric anisotropy in concrete aggregates. While the approach shows promising accuracy, several limitations should be acknowledged. First, the present work does not yet establish a direct quantitative relationship between the proposed anisotropy index and macroscopic mechanical properties such as compressive strength or tensile strength. Second, although the SAM-based segmentation achieved encouraging performance (*F*_1_-*score* of 0.842 and *IoU* of 0.739), further improvements remain necessary, particularly through domain-specific fine-tuning to capture smaller or more irregular aggregates. Third, the dataset employed for validation consists of 108 images, which, although diverse in aggregate size, is relatively limited in scope; broader validation on larger and more varied datasets will be essential to ensure robustness and generalizability. Finally, the present analysis is restricted to two-dimensional cross-sections, whereas three-dimensional CT-based reconstruction would provide a more comprehensive view of aggregate orientation.

Future work would address these limitations regarding several aspects. Controlled laboratory experiments and numerical simulations could be conducted to establish explicit correlations between the anisotropy index and the mechanical performance of concrete. A larger and more diverse dataset could be constructed, and domain-adapted fine-tuning of the SAM could be explored to further improve segmentation precision. In parallel, systematic benchmarking against supervised deep learning algorithms, such as U-Net, Mask R-CNN, and YOLO, could be performed to evaluate trade-offs in the accuracy, training cost, and deployment practicality. Finally, extending the framework to three-dimensional imaging modalities would enable volumetric characterization of anisotropy and open the way for anisotropy-aware mixture design and intelligent process control strategies for enhanced structural performance.

Meanwhile, the proposed research offers demonstrable translational value for practical engineering. By quantifying the orientation and anisotropic distribution of concrete aggregates, the findings provide a scientific basis for optimizing the mix design in high-performance concrete, particularly for critical structures, such as dams, bridges, and high-rise buildings, where load-bearing reliability is paramount. The analysis of aggregate directionality also contributes to predicting crack propagation paths and durability risks, thereby supporting long-term service performance evaluation. From a construction perspective, understanding how compaction or vibration processes induce aggregate alignment offers practical guidance for optimizing field techniques and mitigating potential weak planes. Furthermore, the proposed approach can be integrated with non-destructive testing methods such as CT scanning, ultrasound, or image-based inspection for quality assessment and hidden defect diagnosis. It may also inform emerging construction practices, including 3D-printed concrete, by improving structural homogeneity and mechanical consistency.

## 5. Conclusions

Quantifying the fabric anisotropy of concrete aggregate is critical for understanding concrete microstructure and predicting mechanical behavior. The accurate segmentation of concrete aggregates is essential to quantify their fabric anisotropy. This study introduces the Segment Anything Model (SAM) for automated concrete aggregate segmentation and proposes a computational geometry-based method combined with second-order Fourier series to quantify fabric anisotropy and dominant orientation. The following main conclusions were drawn:

(1) The SAM achieves effective zero-shot segmentation of concrete aggregates without additional training. On the self-constructed dataset, SAM achieved a *Precision* of 0.894, *Recall* of 0.796, *F*_1_-*score* of 0.842, and *IoU* of 0.739, demonstrating a high accuracy and robustness.
(2) The computational geometry approach combined with second-order Fourier series provides accurate assessment of the aggregate orientation and fabric anisotropy. The average absolute discrepancies in directional and fabric anisotropy indicators are 4.15° and 0.025, respectively, validating the reliability of the proposed method.
(3) The segmentation performance is sensitive to the number of SAM grid points. Increasing grid points shifts the results from under-segmentation to over-segmentation, with an optimal performance observed at 32 grid points.

Future work can be conducted on enhancing the ability of SAM to differentiate multiple target types and on validating the geometry- and Fourier-based quantification across diverse concrete compositions and loading conditions.

## Figures and Tables

**Table 1:** The mix proportion of concrete specimens.

Cement (kg/m^3^)	Sand (kg/m^3^)	Aggregate (kg/m^3^)		Water (kg/m^3^)	Fly Ash (kg/m^3^)	Admixture (kg/m^3^)	Slag (kg/m^3^)
		Fine (5–10 mm)	Coarse (10–20 mm)				
190	752	849	284	95	130	5	80

**Table 2:** Segmentation performance of different methods for concrete aggregate.

Method	*Precision*	*Recall*	*F*_1_-*Score*	*IoU*
Otsu threshold	0.765	0.748	0.756	0.611
Iterative threshold	0.919	0.505	0.652	0.486
SAM	0.894	0.796	0.842	0.739

**Table 3:** Segmentation performance of different grid points.

Point Number	*Precision*	*Recall*	*F*_1_-*Score*	*IoU*	Time
2	0.876	0.042	0.087	0.042	6.20
4	**0.954**	0.116	0.204	0.115	6.54
8	0.938	0.399	0.558	0.389	8.04
16	0.900	0.711	0.794	0.658	12.31
32	0.848	0.843	**0.845**	**0.732**	24.98
64	0.797	0.852	0.823	0.700	47.24
128	0.780	**0.854**	0.815	0.688	135.03

**Table 4:** Segmentation results of SAM-based concrete aggregate.

Image	*Precision*	*Recall*	*F*_1_-*Score*	*IoU*
Image No. 1	0.919	0.849	0.883	0.791
Image No. 2	0.912	0.818	0.862	0.758
Image No. 3	0.93	0.823	0.873	0.775
Image No. 4	0.913	0.867	0.889	0.801
Average	0.919	0.839	0.877	0.781

**Table 5:** Anisotropy evaluation results of concrete aggregate.

Image	Orientation Evaluation			Anisotropy Evaluation		
	Manual	Proposed Method	Absolute Error	Manual	Proposed Method	Absolute Error
Image No. 1	66.0°	72.3°	6.3°	0.141	0.147	0.006
Image No. 2	48.9°	51.4°	2.5°	0.138	0.175	0.027
Image No. 3	77.5°	71.9°	5.6°	0.100	0.071	0.029
Image No. 4	155.0°	157.2°	2.2°	0.128	0.089	0.039
Average	-	-	4.15°	-	-	0.025
Variance	-	-	3.313°	-	-	0.000144

## Data Availability

The data that support the findings of this study are available from the corresponding author.
